# Supplementary material for: Targeting the Melanocortin 1 Receptor in Melanoma: Biological Activity of α-MSH–Peptide Conjugates
Source: Int J Mol Sci. 2024 Jan 16;25(2):1095. doi: 10.3390/ijms25021095 (PMC10816934; doi:10.3390/ijms25021095)

Targeting the melanocortin 1 receptor in melanoma: biological activity of α-MSH peptide conjugates

Ildikó Szabó*. Beáta Biri-Kovács. Ivan Ranđelović. Balázs Vári. Diána Vári-Mező. Szilvia Bősze. József Tóvári. Gábor Mező*

*Correspondence to: Dr. Ildikó Szabó. Research Group of Peptide Chemistry. Eötvös L. Research Network. Eötvös L. University. 1518 Budapest. Pázmány P. stny. 1/A. Hungary; Tel.: (+36)-1-209-0555/1409; Fax: (+36)-1-372-2620; e-mail: ildiko.szabo@ttk.elte.hu and Prof. Dr. Gábor Mező. Research Group of Peptide Chemistry. Eötvös L. Research Network. Eötvös L. University. 1117 Budapest. Pázmány P. stny. 1/A. Hungary; Tel.: (+36)-1-209-0555/1433; Fax: (+36)-1-372-2620; e-mail: gabor.mezo@ttk.elte.hu

Table of Contents:

Materials for peptide synthesis and conjugation……………..S2

HPLC chromatogram and mass spectra of **Conj1**………………………….…………...………………...S3

HPLC chromatogram and mass spectra of **Conj2**……………………………..S4

HPLC chromatogram and mass spectra of **Conj3**…...……………………….……………………………S5

HPLC chromatogram and mass spectra of **Conj4**..S6

Acute toxicity of **Conj2** on BALB/c………………………………………………………………………S7

In vivo antitumor effect of **Conj1**. **2**. **3** and free Dau in B16 murine melanoma bearing C57BL/6 mice………...……………………………………………………………………………………….S8

Toxicity studies measured by liver weight after *in vivo* treatment…………………………………………S9

Acute toxicity of **Conj2** and **Conj4** male NOD-SCID mice……………………………………………..S10

Materials for peptide synthesis and conjugation

Amino acid derivatives and resins were obtained from Iris Biotech (Marktredwitz. Germany). Reagents. such as N.N′-diisopropylcarbodiimide (DIC). triisopropylsilane (TIS). 1-hydroxybenzotriazole (HOBt). 1.8-diazabicyclo[5.4.0]undec-7-ene (DBU) were purchased from Sigma (Budapest. Hungary). Trifluoroacetic acid (TFA) and acetonitrile (AcN) were from VWR (Budapest. Hungary). N.N-dimethylformamide (DMF). dichlormethane (DCM). diethyl ether. and ethanol were purchased from Reanal (Budapest. Hungary)

Figure S1-S4. HPLC chromatogram and mass spectra of Conj1-Conj4

The retention time was obtained on a Phenomenex Jupiter C12 column with the applied linear gradient. The peak detection was measured at $\lambda = 214$ nm. The MS spectrum was measured on a Thermo Scientific Q Exactive Focus Hybrid Quadrupole-Orbitrap Mass Spectrometer. Detailed instrument and method parameters were described in the methods section (S3-S4).

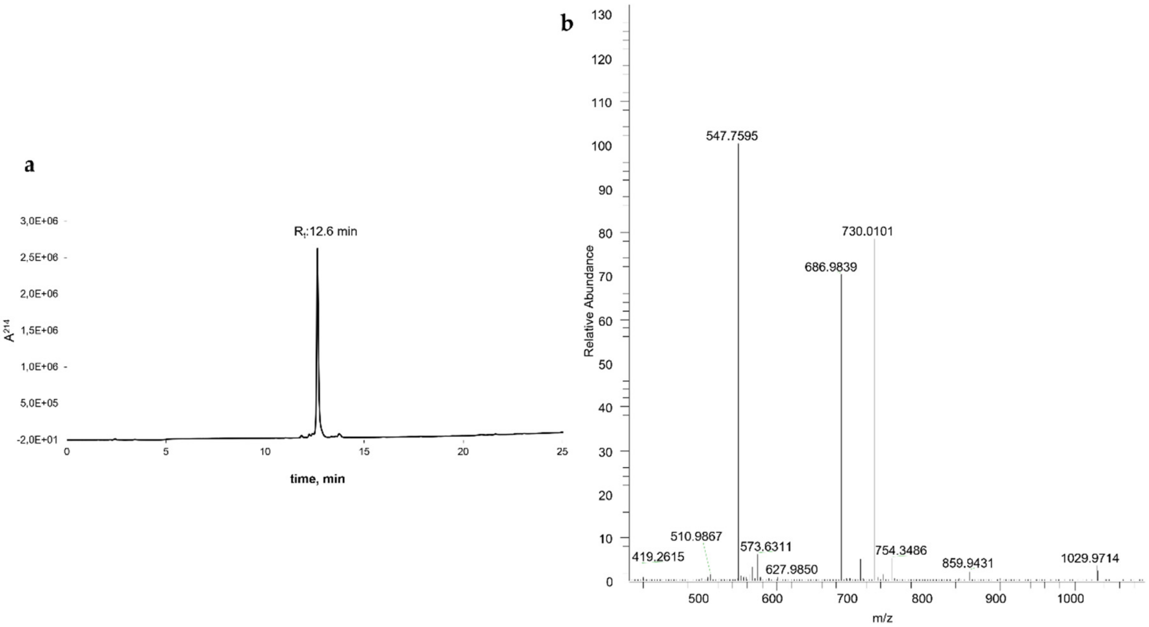

Figure S1. RP-HPLC chromatogram (**a**) and ESI-HRMS spectrum (**b**) of **Conj1**

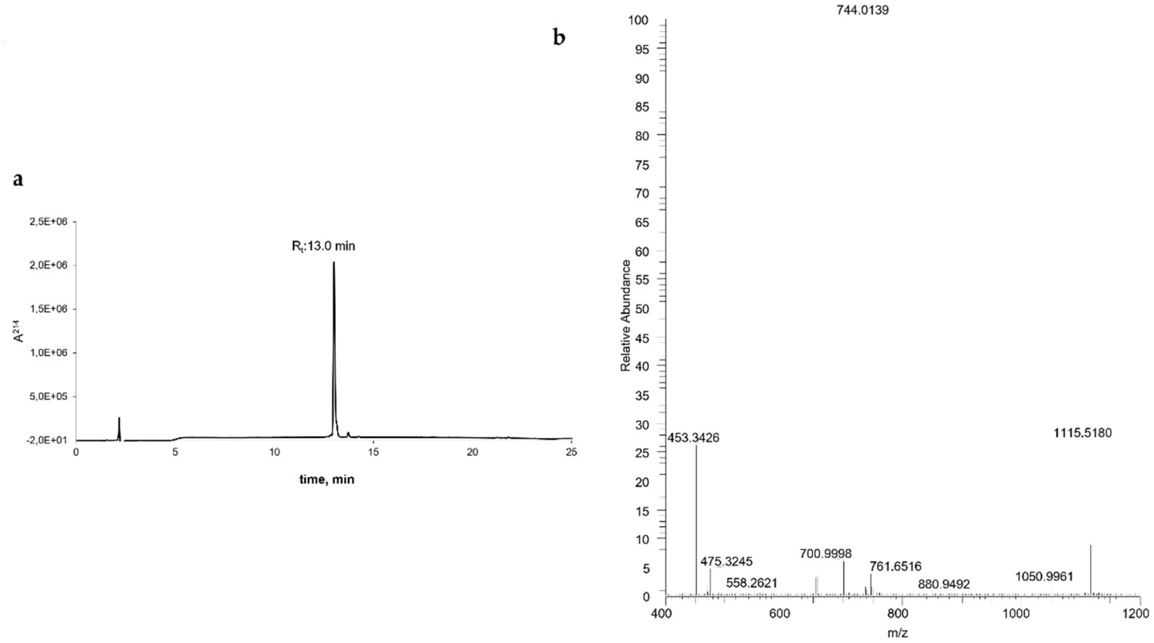

Figure S2. RP-HPLC chromatogram (**a**) and ESI-HRMS spectrum (**b**) of **Conj2**

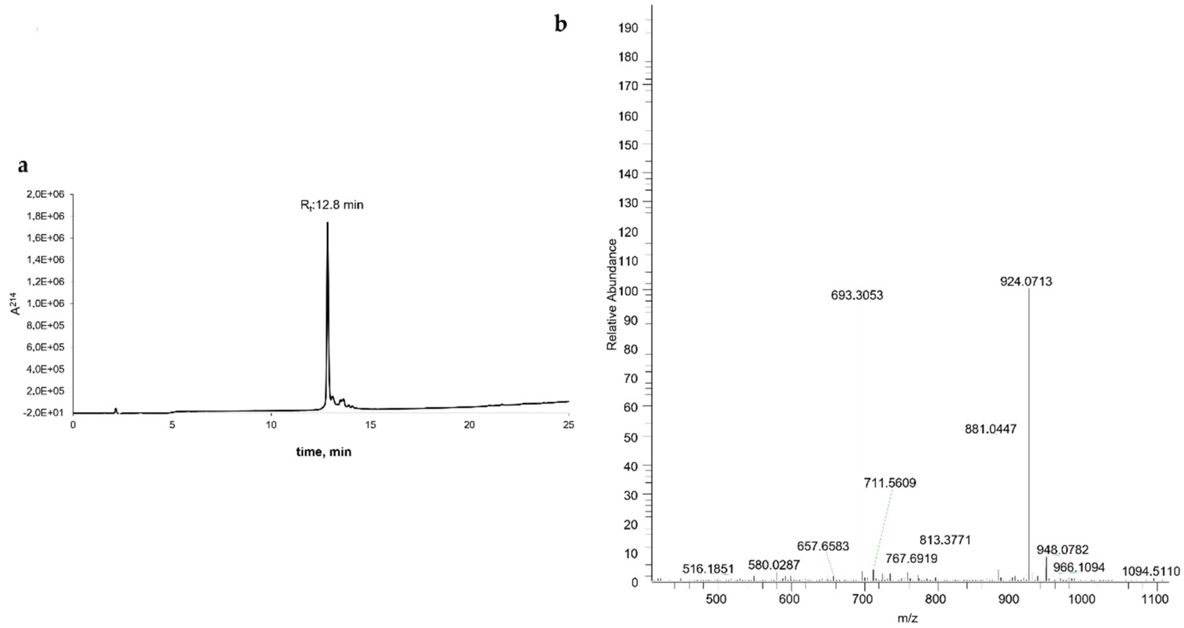

Figure S3. RP-HPLC chromatogram (**a**) and ESI-HRMS spectrum (**b**) of **Conj3**

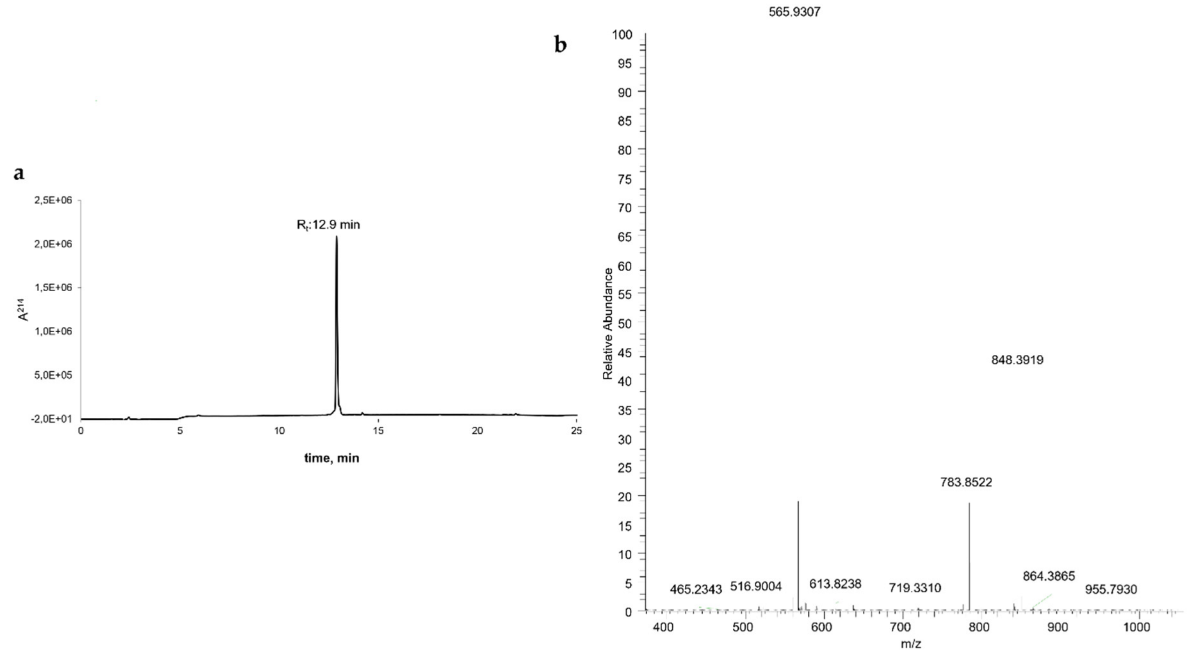

Figure S4. RP-HPLC chromatogram (**a**) and ESI-HRMS spectrum (**b**) of **Conj4**

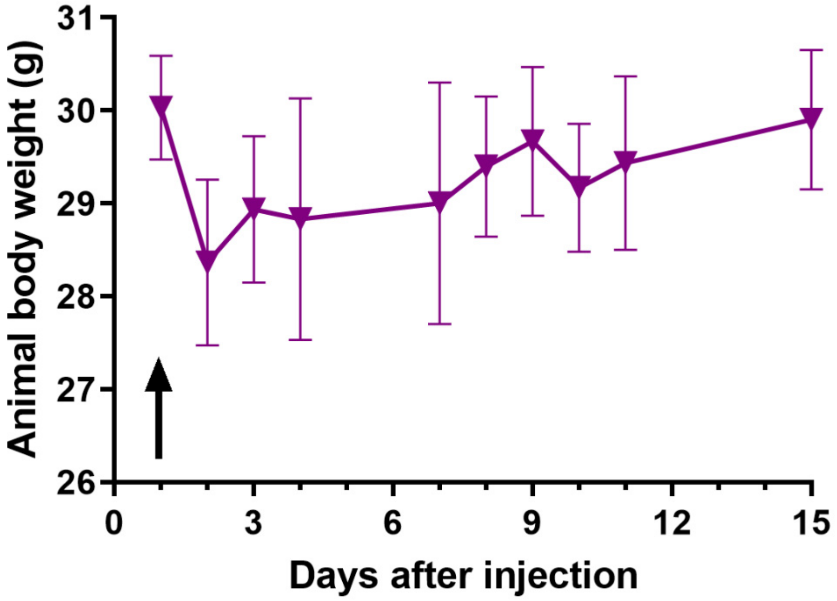

Figure S5. Acute toxicity of Conj2 on healthy male BALB/c mice. Animal body weight (grams. average ± SEM) in in vivo acute toxicity study of conjugate **Conj2** under dose of 25 mg/kg Dau content. ↑: treatment. 3 animals

Table S1. Effect of Dau-conjugates (10 mg Dau content/kg) and free Dau (1 mg /kg) in subcutaneous B16 murine melanoma bearing C57BL/6 male mice *in vivo*.

**Parameter**	**Treatment**				
	**Control**	**Dau**	**Conj1**	**Conj2**	**Conj3**
**Animal body weight**	+9.4	+7.6	+12.9	+8.5	+18.2
**Tumor volume in mm^3**		-4.9	-23.8	-37.8	+12.3
**Tumor volume in %**		+58.8	+9.7	-75.4	-14.7
**Tumor weigh in %**		-17.0	-2.4	-38.6	+9.2

Values represent percentage (%) where minus means decreasing of animal body weight at the end of experiment compared to the start. inhibition of tumor compared to control group. decreasing of tumor weight compared to control group.

Table S2. Toxicity studies measured by liver weight after *in vivo* treatment.

	Normalized liver weight			
	**Control**	**Dau**	**Conj2**	**Conj4**
	4.93%	4.05%	3.77%	5.23%
	5.37%	4.31%	5.10%	4.34%
	5.74%	N/A	4.35%	4.47%
	4.45%	4.81%	4.14%	4.50%
	5.09%	4.07%	4.73%	5.35%
	5.11%	4.07%	4.15%	4.70%
	4.37%	5.29%	4.90%	5.11%
ave	5.54%	N/A	4.55%	4.62%
relative to ctr	4.66%	5.00%	5.02%	4.52%

Figure S6. Determination of *in vivo* toxicity of **Conj2** and **Conj4** in OCM-1 bearing male NOD-SCID mice

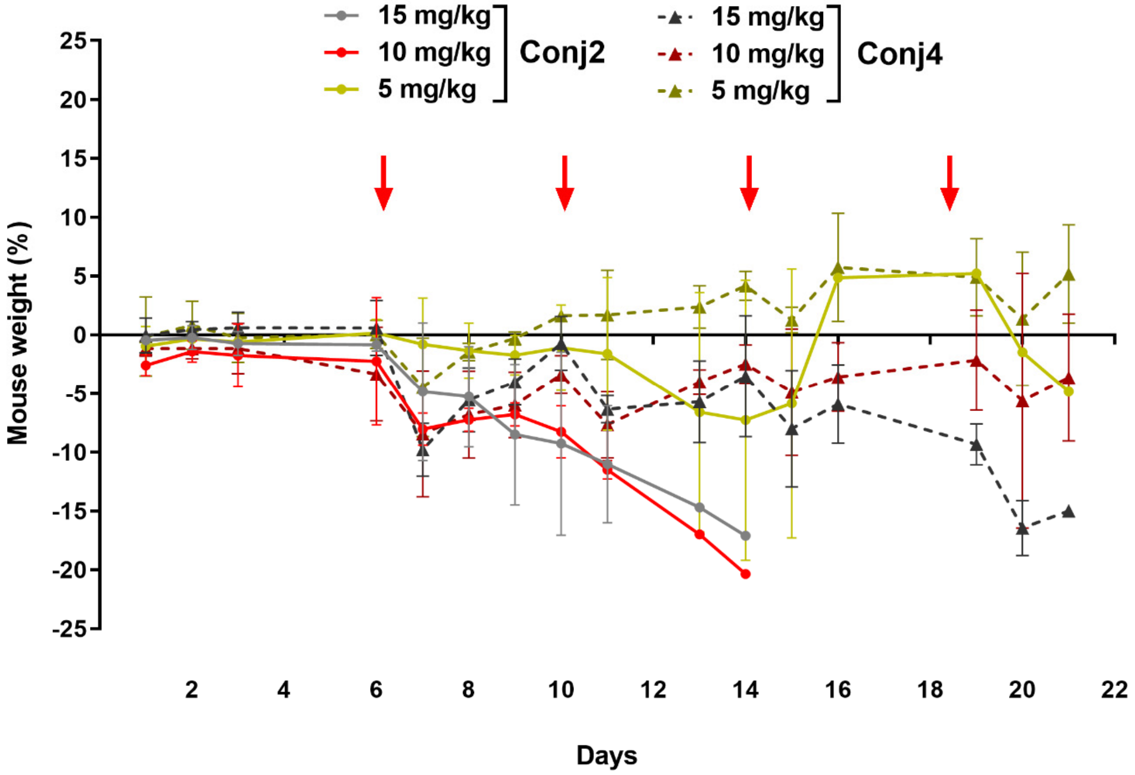

Supplement: Supplementary file 1 [file ijms-25-01095-s001.zip › ijms-2764258-supplementary.pdf]
